# Co-production of Nisin and γ-Aminobutyric Acid by Engineered *Lactococcus lactis* for Potential Application in Food Preservation

**DOI:** 10.3389/fmicb.2020.00049

**Published:** 2020-01-29

**Authors:** Jiaheng Liu, Furong Meng, Yuhui Du, Edwina Nelson, Guangrong Zhao, Hongji Zhu, Qinggele Caiyin, Zhijun Zhang, Jianjun Qiao

**Affiliations:** ^1^Key Laboratory of Systems Bioengineering, Ministry of Education, Tianjin University, Tianjin, China; ^2^School of Chemical Engineering and Technology, Tianjin University, Tianjin, China; ^3^SynBio Research Platform, Collaborative Innovation Center of Chemical Science and Engineering, Tianjin, China; ^4^Key Laboratory of Molecular Medicine and Biotherapy, School of Life Sciences, Beijing Institute of Technology, Beijing, China; ^5^Key Laboratory of Storage of Agricultural Products, Ministry of Agriculture and Rural Affairs, Tianjin, China

**Keywords:** *Lactococcus lactis*, nisin, GABA, metabolic engineering, food preservative

## Abstract

Microbiological contamination and oxidative damage are the two main challenges in maintaining quality and improving shelf-life of foods. Here, we developed a *Lactococcus lactis* fermentation system that could simultaneously produce nisin, an antimicrobial peptide, and γ-aminobutyric acid (GABA), an antioxidant agent. In this system, we metabolically engineered a nisin producing strain *L. lactis* F44 for GABA production by expression of glutamate decarboxylase and glutamate/GABA antiporter. GABA biosynthesis could facilitate nisin production through enhancing acid resistance of the strain. By applying a two-stage pH-control fermentation strategy, the engineered strain yielded up to 9.12 g/L GABA, which was 2.2 times higher than that of pH-constant fermentation. Furthermore, we demonstrated the potential application of the freeze-dried fermentation product as a preservative to improve the storage performance of meat and fruit. These results suggested that the fermentation product of nisin–GABA co-producing strain could serve as a cost-effective, easily prepared, and high-performance food preservative.

## Introduction

Microbiological contamination is a crucial issue in food preservation resulting in the alteration of food qualities including nutritional value, flavor, and tastes (Chatterjee and Abraham, 2018). More seriously, an increase of foodborne diseases caused by foodborne pathogens has been reported (Fleetwood et al., 2019). Synthetic preservatives, including sodium benzoate, potassium sorbate, sodium lactate, methylparaben, propylparaben and butylated hydroxyanisole (BHA), have a long and impressive history in food preservation. However, some of them, such as BHA or butylated hydroxytoluene (BHT), are suspected to present toxicity effects to humans (Bauer et al., 2001; de Oliveira Pateis et al., 2018). Therefore, their potential health threats to humans have prompted investigations into applying natural preservatives in recent years (Przybylski et al., 2016). Nisin, a natural antimicrobial peptide, exhibits broad-spectrum antimicrobial activity against many of the Gram-positive foodborne bacteria, such as *Listeria* and *Clostridium* (Cotter et al., 2005; Barbosa et al., 2017). It also exhibits antimicrobial activity against some Gram-negative bacteria such as *Escherichia coli* and *Salmonella* spp. when combined with ethylenediaminetetraacetic acid (EDTA) or other treatments, such as heat and physical treatment (Belfiore et al., 2007; Gharsallaoui et al., 2016). It has been widely applied to prevent microbial growth in food products, such as cheese (Kallinteri et al., 2013; Cui et al., 2016), sausage (Wijnker et al., 2011; Araújo et al., 2018), meat (Solomakos et al., 2008; Ferrocino et al., 2015), and fruit juice (de Oliveira Junior et al., 2015; Song et al., 2019).

Nisin is industrially produced by certain strains of *L. lactis* subsp. *lactis*, a food-grade microorganism with a long history in industrial dairy fermentations. Currently, one major problem in nisin production is that nisin separation and purification are time-consuming and not cost-effective. Thus, pure product of nisin (>99%) is commercially unavailable (Juturu and Wu, 2018). The commercial nisin preparation called Nisaplin is a semi-product of nisin, which contains 2.5% (w/w) nisin, and the rest are mainly NaCl and proteins from the culture medium. In some cases, the nisin fermentation broth is immediately applied in food preservation, which reduces the cost at the expense of less preservative effectiveness due to the bounding effect between nisin and proteins (Cleveland et al., 2002).

Oxidation of lipids, proteins, and carbohydrates is another critical factor that results in deterioration of food quality and shortening of shelf life (Di Bernardini et al., 2011). As a ubiquitous four-carbon, non-protein amino acid, γ-aminobutyric acid (GABA) has proved to be effective in inhibiting the formation of lipoxidation end-products due to its scavenging effects on reactive carbonyl compounds (Deng et al., 2010). GABA has been generally recognized as safe (GRAS) by the Food and Drug Administration. Japan first introduced GABA into foodstuff market due to its physiological functions (Jiang et al., 2010). Furthermore, GABA can alleviate oxidative damage of fruits (Yang et al., 2011; Li et al., 2019) and barley seedlings (Song et al., 2010) through enhancing the activities of antioxidant enzymes. Exogenous GABA treatment significantly improves the storage performance of fruits through increasing the accumulation of citrate and amino acids (Sheng et al., 2017), reducing chilling injury (Shang et al., 2011), and altering the carbon and nitrogen metabolism (Zhu et al., 2019). Therefore, GABA has great potential as an antioxidant or additive in food preservation.

Recently, many researchers have focused on efficient production of GABA using lactic acid bacteria (LAB), mainly *Lactobacillus brevis*, as microbial cell factory due to the application of GABA in food, pharmaceutical, and agricultural industries and the GRAS feature of LAB (Wu and Shah, 2017). Although *L. lactis* subsp. *lactis* generally possesses a complete GABA biosynthesis pathway, which can even be regarded as a characteristic to distinguish it from other *L. lactis* subspecies (Nomura et al., 1999), only a few *L. lactis* strains are reported to have a relatively higher production of GABA (Diana et al., 2014; Oliveira et al., 2014; Laroute et al., 2016).

In this study, we propose that the fermentation broth of a nisin and GABA co-producing strain would be a good preservative candidate which could exhibit both antimicrobial and antioxidative activities, leading to a better preservation performance than nisin fermentation broth. Although there have been many reports for individual production of nisin and GABA by *L. lactis*, research into their combined production has not been reported. Therefore, the objective of this study is to construct a nisin and GABA co-producing *L. lactis* strain and evaluate the efficacy of the application of the fermentation product in food preservation.

## Materials and Methods

### Strains, Media, and Culture Conditions

All bacterial strains used in this study were listed in Supplementary Table S1. The parent strain was *L. lactis* F44, a nisin producing strain, which was constructed through genome shuffling of *L. lactis* YF11 (accession number CGMCC7.52) in our previous study (Zhang Y.F. et al., 2014). *E. coli* MG1655, used for genomic DNA isolation and gene cloning, was cultured in LB medium. *Micrococcus flavus* ATCC 10240, used as an indicator strain for the bioassay of nisin, was grown in LB medium. Its agar diffusion bioassay medium (pH 7.0) contained (per liter) 8 g tryptone, 5 g glucose, 5 g yeast extract, 5 g NaCl, 2 g Na_2_HPO_4_, and 15 g agar. *L. lactis* F44 and the engineered strains were cultured in 100 mL seed medium (pH 7.2) containing (per liter) 15 g glucose, 15 g peptone, 15 g yeast extract, 20 g KH_2_PO_4_, 1.5 g NaCl, and 0.15 g MgSO_4_⋅7H_2_O. The fermentation medium (pH 7.2) for *L. lactis* strains contained (per liter): 25 g glucose, 15 g peptone, 15 g yeast extract, 20 g KH_2_PO_4_, 1.5 g NaCl and 0.15 g MgSO_4_⋅7H_2_O, 3 g corn steep liquor, and 0.26 g cysteine. The medium for preculture of recombinant *L. lactis* was supplemented with 5 μg/mL erythromycin to maintain plasmid stability. *L. lactis* strain NZ9000, derived from MG1363, lacks the *nis* operon and harbors the regulatory genes *nisR* and *nisK* (Kuipers et al., 1998). NZ9000 was grown in GM17 medium (M17 broth supplemented with 0.5% glucose) at 30°C. All reagents for preparation of the culture media were of analytical grade and purchased from Tianjin Dingguo Biotechnology Co., Ltd. (Tianjin, China).

The flask fermentation was conducted statically in 250 mL flask containing 100 mL fermentation medium supplemented with 5 g/L sodium glutamate and 0.1 mM pyridoxal-5′-phosphate (PLP) at 30°C. The flask fermentation experiments were conducted in triplicate.

Fed-batch fermentation of F44/GadB1C1 was conducted in a 5-L bioreactor (Shanghai Baoxing Bio-Engineering Equipment Co. Ltd., Shanghai, China) containing 2 L fermentation medium supplemented with 20 g/L sodium glutamate and 0.1 mM PLP for 30 h at 30°C, 100 rpm. The glucose concentration was adjusted to about 10 g/L by feeding 25 mL of 800 g/L glucose solution. The broth pH was controlled at 6 or 4.8 by automatically feeding 25% ammonia. Sodium glutamate and PLP were of analytical grade and purchased from Tianjin Dingguo Biotechnology Co., Ltd. (Tianjin, China).

### Construction of Plasmids and Strains

All the plasmids constructed in this study were summarized in Supplementary Table S1. All the primers used for cloning were synthesized in GENEWIZ Inc. (Beijing, China) and listed in Supplementary Table S2. Genomic DNAs were isolated from *L. lactis* F44 and *E. coli* MG1655 using TIANamp Bacteria DNA Kit, purchased from TIANGEN Biotech (Beijing, China). The *gadB* genes of *L. lactis* F44 and *E. coli* MG1655 were amplified from their genomic DNAs by PCR. PCR reaction mixture of 50 μL included 25 μL 2× Phanta Max buffer, 1 μL dNTP mixture (10 mM each), 1 μL template (100 ng/μL), 1 μL forward primer (10 μM), 1 μL reverse primer (10 μM), 1 μL Phanta Max Super-Fidelity DNA polymerase (Vazyme Biotech Co., Ltd., Nanjing, China), and 20 μL ddH_2_O. PCR amplification was performed for 35 cycles. Each cycle consisted of denaturation at 95°C for 30 s, annealing at 55°C for 30 s, and extension at 72°C. The time extension varied with the length of PCR product (1 kb/min). Codon optimization of *gadB* and *gadC* from *Lactobacillus buchneri* WPZ001 was performed on the basis of *L. lactis* usage^1^. The optimized genes were synthesized in GENEWIZ Inc. (Beijing, China). Plasmids were constructed by ligating the target genes into plasmid pLEB124 using EasyGeno Assembly Cloning kit (TIANGEN, Biotech, Beijing, China). The constructed plasmids were verified by PCR amplifications and DNA sequencing. Heat shock transformation was applied to transform the constructed plasmids into *E. coli* TG1 for enrichment. The TG1 competent cells were purchased from TransGen Biotech (Beijing, China). The 10 μL ligated mixture was added to 100 μL competent cells (TG1) and incubated on ice for 30 min. The mixture was then heat-shocked at 42°C for 90 s, and incubated on ice for 5 min. Then 1 mL LB broth was immediately added and incubated at 37°C for 1 h in a shaking incubator. After incubation, a 200 mL aliquot of cell suspension was plated on LB agar containing 200 μg/mL erythromycin and incubated for 24 h. The plasmids were extracted using TIANprep Mini Plasmid Kit (TIANGEN, Biotech, Beijing, China) after antibiotics selection, and then transformed into the *L. lactis* F44 by electroporation transformation. A 1% inoculum of an overnight culture was grown in 100 mL seed medium and 0.1g/mL glycine of 150 ml was added before use at 30°C until the OD_600_ reached 0.5. Ampicillin of 20 μg/mL was added to the medium to weaken the cell wall and cultured at 30°C for 1 h. The cells were harvested and washed three times with sterile buffer containing 0.4 M sucrose and 20% glycerin. For electroporation, 50 μL of the cell suspension was mixed with 400 ng of plasmid DNA and subjected to electroporation at the field strength of 2.5 kV/cm (pulse duration, 5 ms). After the pulse, 950 μL of seed medium was immediately added to the cell suspension, and the sample mixture was incubated for 3 h at 30°C. The transformants were plated onto seed medium agar containing 5 μg/mL erythromycin and incubated for 36–48 h at 30°C.

### Acid Tolerance Assay

Overnight stationary-phase cultures of *L. lactis* F44 and F44/GadB1C1 were harvested by centrifugation at 8,000 rpm for 5 min and washed twice with 0.9% NaCl. Then *L. lactis* was resuspended in an equal volume of fermentation medium adjusted to pH 3.0 with 18% hydrochloric acid and incubated for 3 h under anaerobic condition. Suspension aliquots before and after the pH 3.0 challenge were serially diluted and then plated on seed medium agar plates for colony counting to calculate the survival ratio.

### Preparation of Freeze-Dried Fermentation Product

The 24 h culture of F44 and 26 h culture of F44/GadB1C1 were collected and centrifuged at 8,000 rpm for 5 min. Then the supernatant was filter sterilized through a 0.22-μm syringe filter and moved to a 120-mm plastic petri dish. After froze in −80°C refrigerator, the freeze-dried fermentation product was obtained by drying with an LGJ-10 freeze dryer (Beijing Songyuan Huaxing Technology Development Co., Ltd., Beijing, China). The cold trap temperature was set at −40°C. Then the freeze-dried preparation was stored in a reclosable vacuum storage bag at −20°C for further use.

### Preparation of Pork Samples and Treatments

Fresh lean pork (hind leg) was purchased from a local retail market (Tianjin, China) and then immediately minced in a professional meat grinder (Shangyuan, SYP-MM 12, Guangdong, China). The homogenized pork samples were mixed with 0.1 g/kg of potassium sorbate, 0.1 g/kg F44/GadB1C1 fermentation product, 0.1 g/kg F44 fermentation product, 0.1 g/kg BHA, and 0.2 g/kg BHA, respectively, by blending for 5 min using a Kitchen Aid Mixer (Kitchen Aid, St. Joseph, MI, United States). Then the treated pork samples were stored in a sterile plastic container at 4°C. The sample without any additive was used as control. All the treatments were conducted in duplicate.

### Microbiological Analyses

The microbial analyses were carried out at days 0, 4, 8, and 12 during storage at 4°C. Five grams of pork meat was transferred to a sterile stomacher bag, and 45 mL of saline/peptone water (0.1% peptone and 0.85% NaCl) was added. After 2 min homogenization, 1 mL aliquots (triplicate samples per dose point) from the mixture was 10-fold serially diluted and 100 μL of the appropriate dilutions was spread onto different selective agars including PDA for yeasts and molds counts, VRBA for coliform bacteria counts, and MRS agar for LAB counts. The PDA and MRS agar plates were incubated at 30°C for 72 h, while the VRBA plates at 37°C after 24 h. The results were expressed as log_10_ of the numbers of CFU per gram of pork (log_10_ CFU/g).

### Assessment of Lipid Oxidation

The lipid oxidation of pork was evaluated by detecting the thiobarbituric acid-reactive substances (TBARS) as previously described (Witte et al., 1970). The samples were taken for TBARS analyses at days 0, 2, 4, 6, 8, 10, and 12. Five grams of pork was added to 25 mL of solution containing 7.5% trichloroacetic acid and 0.1% EDTA. Then the homogenates were filtered twice with double-layer filter paper to remove grease. Five milliliters of filtrate were transferred into a 25 mL colorimetric tube, and 5 mL thiobarbituric acid solution (0.02 M) was added. Then the mixture was kept in the dark for 17 h at room temperature. The resulting colors were read at the absorbance of 530 nm with a TU-1810 spectrophotometer (Purkinje General Instrument Co., Ltd., Beijing, China), and the results were expressed as micrograms of malondialdehyde (MDA) per gram of pork.

### Preparation of Strawberry Samples and Treatments

Strawberries were picked up from a packing house in Tianjin, China. The strawberries free from visible damage and without significant differences in size and color were selected for further preservation experiments. Then 180 strawberries were randomly and equally divided into six groups, which were dipped in sterile water (as control), 0.1% sodium benzoate solution, 0.1% potassium sorbate solution, and 0.1% solutions of freeze-dried products of F44, F44/GadB1C1, and NZ9000 fermentation broth for 2 min, respectively. After air drying in a sterile work bench, the strawberries were stored at room temperature with a relative humidity of 50–60% for 5 days. All the treatments were conducted in duplicate.

### Determination of Rotting Rate

The strawberries with appearance of mildew, injury, or black spot were considered to be rotten. The number of rotten strawberries was recorded and removed from the samples at days 1, 2, 3, 4, and 5 during the storage. The rotting rate was calculated as the ratio between the number of rotten samples and the initial number of test samples in each group.

### Colorimetric Analysis

Colorimetric analysis was performed on the strawberries with a colorimeter (CR200, Minolta, Osaka, Japan), which gave the results of CIE (International Commission on illumination) *L*, *a*, *b* values according to the CIE 1976 standard. The CIE LCH system was used to evaluate the color change of strawberries during the storage. The values of *C* and *H* represented chroma (saturation) and hue angle, respectively. *C-*value was calculated as (*a*^2^ + *b*^2^)^1/2^, and *h-*value was calculated as arctan *b*/*a*.

### Analytical Methods

Glucose concentration in the fermentation broth was determined using a biosensor analyzer (SBA-40B, Biology Institute of Shandong Academy of Sciences, China). The pH of fermentation broth was measured with a pH meter (FE20, Mettler Toledo, Shanghai, China). The nisin titer assay was performed as described in our previous report (Liu et al., 2017). Nisin standard was purchased from Sigma Chemical Company (Shanghai, China). The high-purity nisin (>99.5%) was kindly provided by Chihon Biotechnology Co., Ltd. (Luoyang, China).

Extracellular concentration of glutamate and GABA was determined by online pre-column derivatization RP-HPLC (Waters 600 series, Waters Corporation, Milford, MA, United States), equipped with a Waters 2996 Photodiode Array Detector and an Ultimate^®^ Amino Acid column (4.6 mm × 250 mm, 3.5 μm, Welch Materials, Shanghai, China). Two mobile phases including A: 0.1 M sodium acetarsenate (pH 6.5):acetonitrile (93:7) and B: water:acetonitrile (20:80) were applied. The processes of derivatization and determination were recommended by the supplier (Welch Materials, Shanghai, China^2^). The standard of glutamate and GABA was purchased from Sigma Chemical Company (Shanghai, China).

## Results and Discussion

### Construction of Nisin and GABA Co-producing *L. lactis* Strain

**FIGURE 1:**
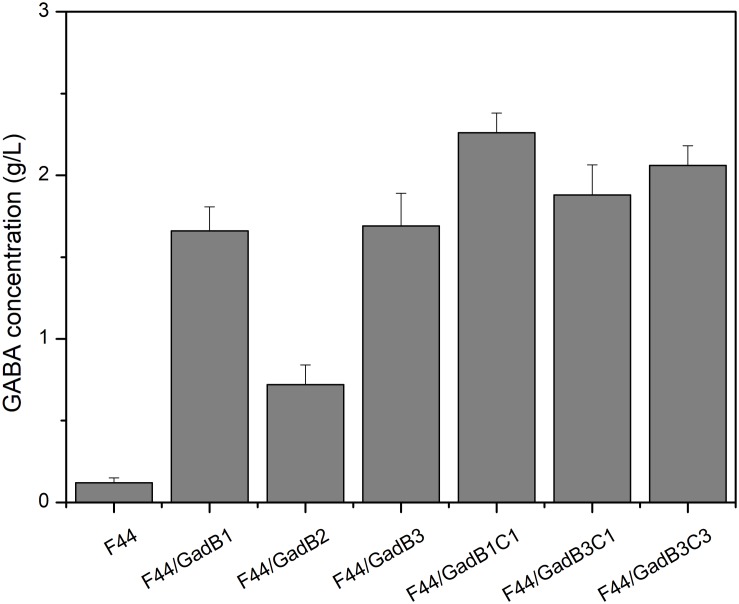
Comparison of GABA production obtained at 24 h by batch cultivation of the original strain F44 and the engineered strains.

*Lactococcus lactis* F44, exhibiting high production of nisin, was used to construct nisin and GABA co-producing strain in this research. GABA is synthesized from glutamate in a decarboxylation reaction catalyzed by glutamate decarboxylase (GAD), and PLP is an essential cofactor for the biotransformation process (Xu et al., 2017). Therefore, we evaluated the GABA-producing capacity of F44 in fermentation medium supplemented with 5 g/L sodium glutamate and 0.1 mM PLP. Only 0.12 g/L GABA was detected after 24 h fermentation, indicating this original strain cannot be used in industrialized production of GABA. Construction of nisin and GABA co-producing strain was initiated with enhancing GAD activity. Three GAD-encoding gene, including *gadB* from *L. lactis* F44, *gadB* from *E. coli* MG1655, and codon-optimized *gadB* from a GABA-producing LAB, *Lb. buchneri* WPZ001 (Zhao et al., 2015), were cloned or synthesized, and expressed in F44 using plasmid pLEB124 under the control of a strong and constitutive promoter P45, generating strains F44/GadB1, F44/GadB2, and F44/GadB3, respectively. As shown in Figure 1 and Supplementary Figure S1, all the three recombinant strains showed remarkable increases in production rate of GABA. Significantly, the GABA concentration after 24 h culture of F44/GadB1 and F44/GadB3 reached 1.66 and 1.69 g/L, which was about 14 times higher than that of F44. In *L. lactis*, glutamate/GABA antiporter, encoded by *gadC*, is responsible for pumping GABA out of the cell, at the same time, bringing in glutamate. To further enhance the GABA production capacity, *gadC* was overexpressed in F44/GadB1 and F44/GadB3 to make strain F44/GadB1C1 and F44/GadB3C1. As we intended, a higher glutamate production of 2.26 g/L was attained with F44/GadB1C1, and the conversion rate of sodium glutamate reached 84.2%. In addition, we also introduced codon-optimized *gadC* gene from *Lb. buchneri* WPZ001 into F44/GadB3. The resulting strain F44/GadB3C3 produced 2.06 g/L glutamate, which was lower than F44/GadB1C1.

### Enhanced Acid Resistance and Nisin Production in the Recombinant *L. lactis* Strain

**FIGURE 2:**
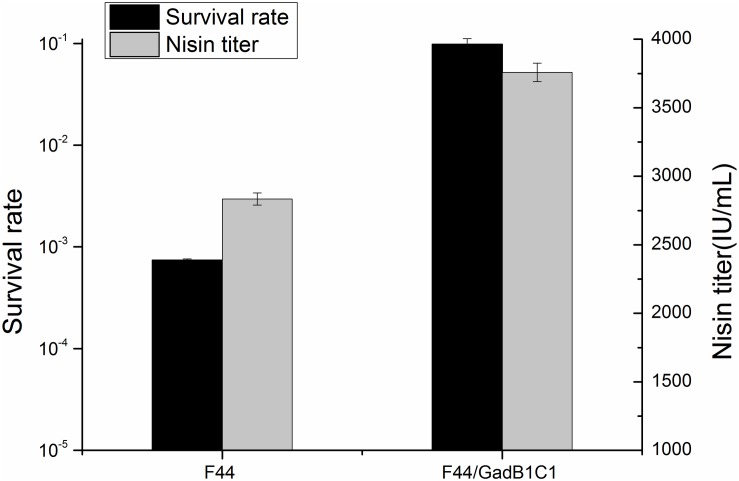
Maximum nisin titer of F44 and F44/GadB1C1 in flask fermentation and their survival rate after 3 h exposure to pH 3.0.

Glutamate decarboxylase pathway, as one of the classical mechanisms for acid resistance of LAB, plays a crucial role in consuming proton and increasing cytoplasmic pH (Papadimitriou et al., 2016). To examine whether overexpression of GadB and GadC had a promoting effect on acid resistance of *L. lactis* F44, the survival rates of F44 and F44/GadB1C1 after 3 h exposure to pH 3.0 in seed medium were analyzed. As expected, a 130-fold increase of survival rate was shown for F44/GadB1C1 compared with F44 (Figure 2). Indeed, previous researches proved that improving the resistance to acidic conditions in the fermentation medium was an effective strategy to increase nisin production (Zhang et al., 2016; Hao et al., 2017). The maximum nisin titer of F44/GadB1C1 reached 3758 IU/mL, which was 33% higher than that of F44.

### Two-Stage pH-Control Fermentation Promoted GABA Accumulation of F44/GadB1C1

**FIGURE 3:**
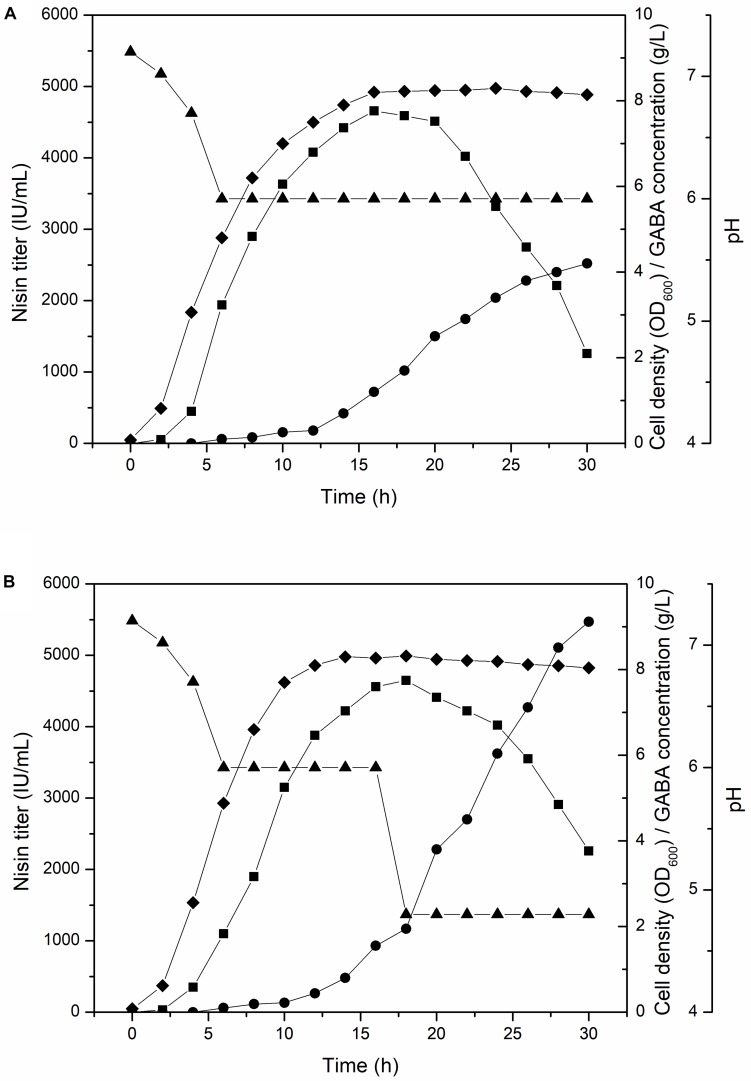
Time profile of cell density (diamond), nisin titer (square), GABA concentration (circle), and pH (triangle) of F44/GadB1C1 in 5-L fermentor using different pH control strategy. **(A)** Maintaining pH 6 and **(B)** adjusting pH to 4.8 at 16 h.

To further increase the GABA production, we conducted fed-batch culture experiment of F44/GadB1C1, the GABA, and nisin co-production strain, in a well controlled 5-L fermenter with a working volume of 2 L at 30°C. The pH of traditional fermentation process for nisin production is controlled at 6, which is optimum for *L. lactis* growth. Maintaining pH constant at 6, the nisin titer increased rapidly from the beginning of fermentation and the maximum nisin titer of 4660 IU/mL was attained at 16 h. After 20 h, there is an obvious decrease in the nisin titer and only 1461 IU/mL of nisin was detected at 30 h. However, GABA started accumulating after 12 h and its production reached 4.2 g/L at 30 h (Figure 3A). Indeed, the condition of pH 6 might be unfavorable to the GadB activity and GABA production. It has been reported that GadB exhibited a higher enzyme activity under relatively acidic conditions and a lower pH could increase the GABA yield (Seo et al., 2013; Zhang R. et al., 2014; Shi et al., 2017). Therefore, we compared the conversion rate from sodium glutamate to GABA of strain F44/GadB1C1 at different pH values. About 3 × 10^8^ F44/GadB1C1 cells were collected and cultured in fresh fermentation medium with 1.0 g/L sodium glutamate and 0.1 mM PLP in the range of pH 4.0–6.0 for 0.5 h. The highest conversion rate of 64.5% was occurred at pH 4.8 (Supplementary Figure S2). Although GadB exhibits a higher enzyme activity under relatively acidic conditions, the degree of GABA conversion is dependent on the amount of *L. lactis* cells. Since the optimum neutral pH (6.0–7.0) for *L. lactis* growth is a prerequisite to realize an ideal cell density, we controlled the broth pH at 6.0 for the first 16 h, which could ensure sufficient biomass accumulation and nisin production. Then the pH value was adjusted to 4.8 to facilitate the conversion of glutamate to GABA. By applying this two-stage pH-control approach, the GABA yield increased to 9.12 g/L after 30 h, which was 2.2 times of that at a constant pH of 6 (Figure 3B). The conversion rate of sodium glutamate reached 74.4%. Another benefit of adjusting the broth pH to 4.8 after 16 h was that it obviously reduced the decreasing rate of nisin titer, presumably due to the higher stability and solubility of nisin at lower pH (Penna et al., 2005).

An interesting phenomenon, as shown in the time profiles of nisin titer and GABA concentration, was that the major production periods of nisin and GABA were uncoupled. Since GABA mainly accumulated in the late fermentation stage while nisin titer decreased gradually, it was impossible to simultaneously maximize the production of both nisin and GABA. To the best of our knowledge, the most promising approach to alleviate this problem was expanding the functional pH range of GadB through directed evolution. A GadB mutant, which was active under pH 6, might shorten the fermentation time and reduce the cost of co-producing nisin and GABA.

Due to the relatively high level of both nisin titer and GABA concentration, the fermentation broth at 26 h after removal of *L. lactis* cells by centrifugation and filtration was freeze-dried. For 100 mL fermentation broth, 2.1 g freeze-dried product was obtained (Supplementary Figure S3). The nisin titer and GABA concentration were 1.42 × 10^5^ IU and 0.31 g per gram freeze-dried product, suggesting a 15% loss rates of nisin and an 8% loss rates of GABA after freeze drying, respectively. After 1-month storage at −20°C, only a slight decrease in nisin titer (6%) and GABA concentration (0.4%) was observed, implying that the freeze-dried product was stable. Since both nisin and GABA were GRAS compounds, this freeze-dried fermentation product containing no genetically modified organism could provide reasonably good security in food preservation.

### Effect of F44/GadB1C1 Fermentation Product on Microbiological Growths and Lipid Oxidation in Pork During Cold Storage

**FIGURE 4:**
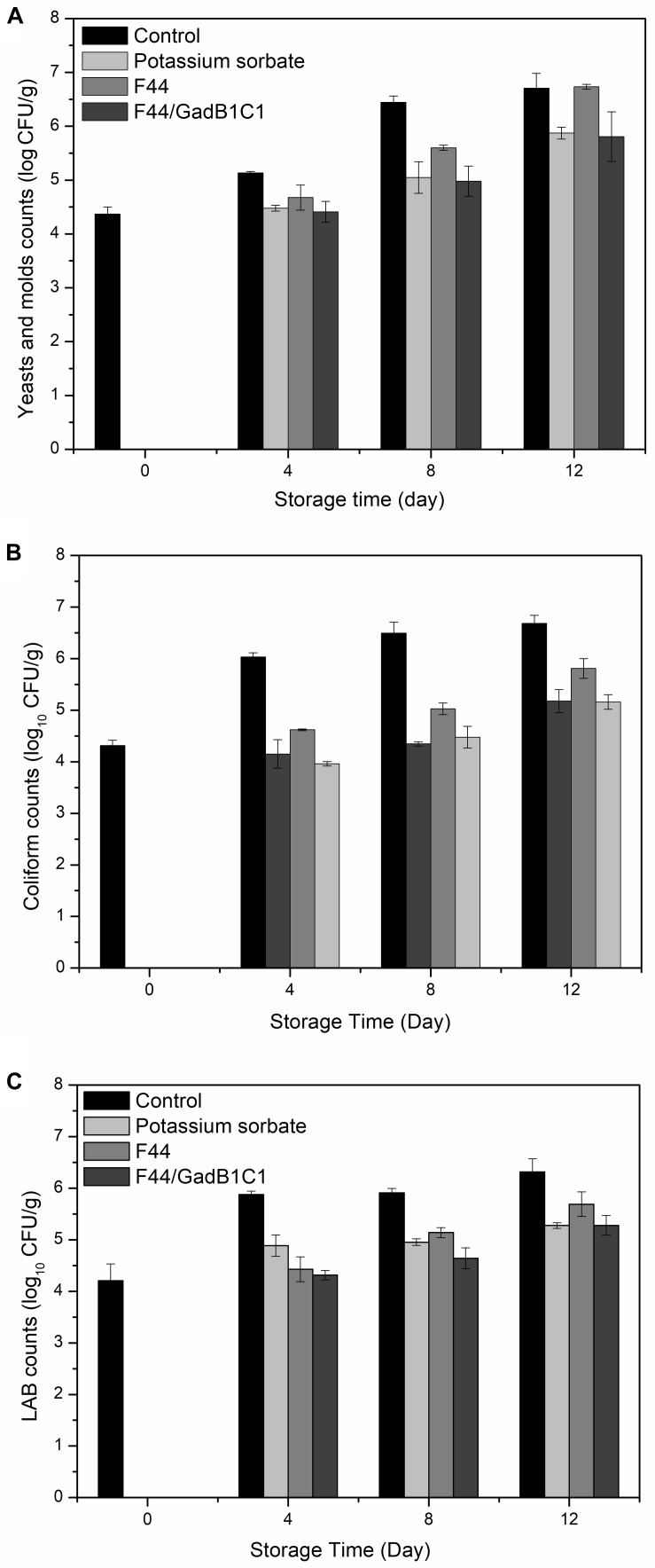
Microbial changes in pork treated with potassium sorbate, F44 and F44/GadB1C1 fermentation product during storage at 4°C. **(A)** Yeasts and molds counts, **(B)** coliform counts, and **(C)** LAB counts.

The main cause of meat rancidity is the growth of microorganisms, such as *E. coli*, *Lactobacillus*, yeast, and fungi. Quantitative microbiological analysis is one of the indicators to assess the spoilage degree of meat. To testify the effect of fermentation product of the nisin and GABA co-producing strain on microbiological growths of meat, we treated pork samples with 0.1 g/kg potassium sorbate, F44/GadB1C1 fermentation product, and F44 fermentation product, respectively. The sample without any treatment was used as control. As shown in Figure 4, all the three groups with additives exhibited a lower microbial population than the control group from day 4 to day 12. The F44/GadB1C1 fermentation product had nearly the same effect as the industrial preservative potassium sorbate in inhibiting yeast and molds. Significantly, the antimicrobial ability of F44/GadB1C1 fermentation product to coliform and LAB was stronger than that of potassium sorbate before day 8. However, there was no significant difference in the counts of both coliform and LAB between the potassium sorbate group and F44/GadB1C1 group at day 12, which might be attributable to the degradation of nisin in F44/GadB1C1 fermentation product. Interestingly, although there was a relatively equal amount of nisin (about 3500 IU/mL) to F44 and F44/GadB1C1 fermentation broth for preparing the fermentation product, treatment with F44/GadB1C1 fermentation product was more effective in inhibiting the microbiological growth. Therefore, it seemed there might be a synergistic effect of nisin and GABA on the antimicrobial property in pork during cold storage. Further studies were needed to elucidate the molecular basis of this phenomena.

**FIGURE 5:**
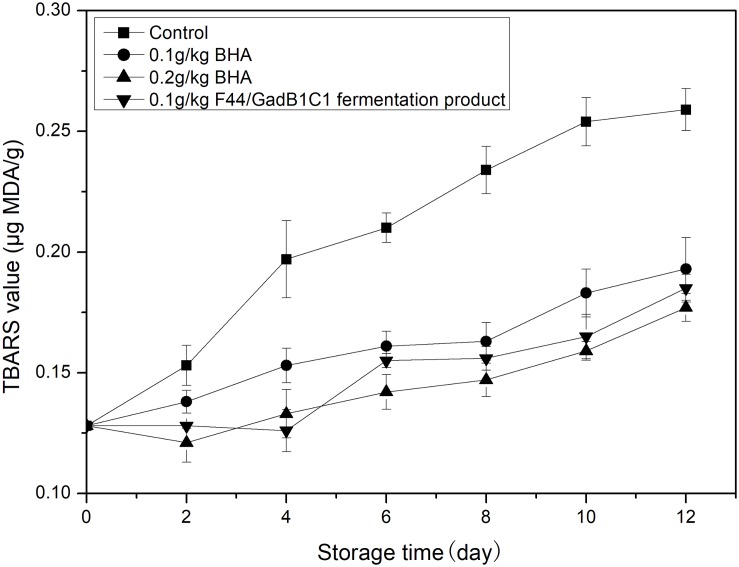
Lipid oxidation evaluation of the pork treated with BHA and F44/GadB1C1 fermentation product during storage at 4°C.

Lipid oxidation is another main factor affecting the overall meat quality, such as flavor, taste, nutritional value, and formation of toxic compounds. The TBARS assay is the most common detection method for evaluating oxidative deterioration. TBARS values are expressed as micrograms of MDA, the main product of lipid oxidation, per gram of sample. Pork samples were treated with 0.1 g/kg of F44/GadB1C1 fermentation product, 0.1 g/kg of BHA, and 0.2 g/kg of BHA, respectively. The untreated pork sample was used as control. The TBARS value of pork was measured for 12 days cold storage (4°C) and the results were shown in Figure 5. The initial TBARS value of the sample was 0.128 μg MDA/g. It obviously increased in all the samples throughout the 12 days, while the increase of the control group was the most striking. After 12 days, the TBARS value of pork treated with 0.1 g/kg F44/GadB1C1 fermentation product was 0.185 μg MDA/g, which was between the value of 0.1 g/kg BHA group (0.193 μg MDA/g) and 0.2 g/kg BHA group (0.177 μg MDA/g). These values were significantly lower than the control sample (0.249 μg MDA/g). It should be noted that the maximum permissible dosage of BHA in meat preservation was 0.2 g/kg and the 0.1 g/kg F44/GadB1C1 fermentation product exhibited a comparable antioxidant capacity. These results indicated that the F44/GadB1C1 fermentation product could be a potential preservative candidate to prevent meat rancidity.

### Effect of F44/GadB1C1 Fermentation Product on Strawberry Rotting Rate and Peel Color

**FIGURE 6:**
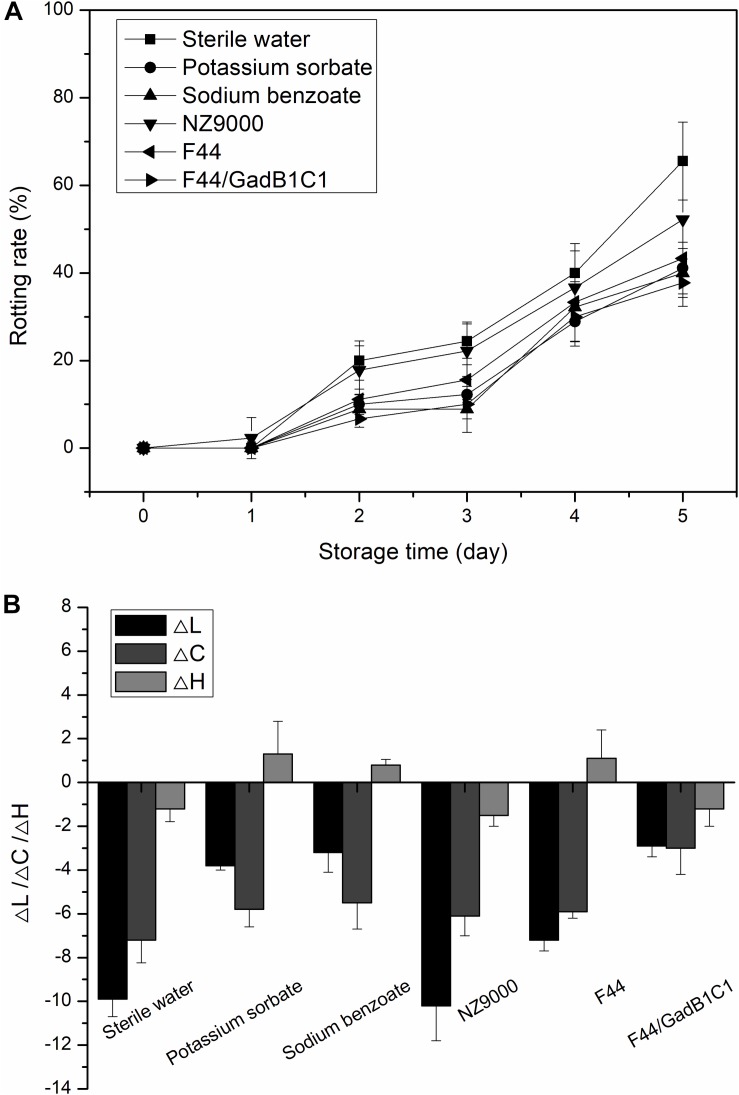
Effect of different treatments on rotting rate **(A)** and color change (Δ*L*, Δ*C*, and Δ*H*) **(B)** of strawberry during storage at room temperature.

We further investigated whether the F44/GadB1C1 fermentation product could improve the storage performance of strawberry. The fresh strawberries were, respectively, dipped in 0.1% (w/w) solutions of F44 freeze-dried product, F44/GadB1C1 freeze-dried product, NZ9000 (a non-nisin-producing and non-GABA-producing *L. lactis* strain), freeze-dried product and sterile water (as control) for 2 min. Sodium benzoate and potassium sorbate, the commonly used commercial preservatives, were also applied to treat the strawberries at the same concentration to provide comparative performance data. Figure 6A showed the changes in rotting rate of strawberry samples within 5 days in each group. The rotting rates of samples treated with F44 and F44/GadB1C1 fermentation products were both lower than that of NZ9000 fermentation products. Significantly, no apparent differences between the samples treated with F44/GadB1C1 fermentation product and the commercial preservatives were observed. It was noteworthy that the strawberry under F44/GadB1C1 freeze-dried product treatment exhibited better storage performance than that with F44 freeze-dried product treatment, which might possibly be associated with the antioxidant effect of GABA on fruit (Yang et al., 2011; Li et al., 2019).

To further evaluate the exact effect of nisin and GABA on storage performance of strawberry, we prepared the solutions of nisin (3.5 mg/L), GABA (310 mg/L), and nisin–GABA mixture at the same concentration to 0.1% solutions of F44/GadB1C1 freeze-dried product, respectively. As shown in Supplementary Figure S4, there was no remarkable difference in the rotting rate between the samples treated with nisin solutions and sterile water. However, the rotting rates of samples treated with F44 and F44/GadB1C1 fermentation products were both lower than that of NZ9000 fermentation products (Figure 6A). We speculated that the improvement of preservation quality of strawberry samples using F44 and F44/GadB1C1 freeze-dried product was due to the synergistic action of nisin and other molecules in the broth, such as lactates, rather than the effect of nisin used alone. Indeed, the use of nisin in combination with other antimicrobial agents such as lactates (Nykänen et al., 2000), lysozyme (Chung and Hancock, 2000), and essential oils (Rohani et al., 2011) has been widely reported to increase nisin activity or even exhibit effectiveness against food-contaminating microorganisms which were not inhibited by nisin alone (Tu and Mustapha, 2002). Both the solutions of GABA and nisin–GABA mixture showed obvious improvements in decreasing the rotting rate of the strawberry samples. This was not unexpected because GABA has been reported to improve the storage performance of fruits through active involvement in the defense against adverse environment in various ways (Sheng et al., 2017).

Then we performed colorimetric analysis to evaluate the peel color change of the strawberries with different treatments storage for 5 days at room temperature (Figure 6B). The analysis was carried out using the CIE LCH system, in which *L*, *C*, and *H* represented lightness, saturation, and hue angle, respectively. To eliminate the impact of differences in the initial LCH values among the different groups, the relative values (Δ*L*, Δ*C*, and Δ*H*) were calculated. The results showed a tendency of a decrease in *L* and *C* values for all the groups. The decline of lightness was considered an unfavorable indicator for strawberry preservation. Compared to the control group, treatment with NZ9000 fermentation product caused a similar change in lightness of strawberry, while the decrease of lightness was slightly alleviated by the treatment of F44 fermentation product. As expected, the treatment of F44/GadB1C1 fermentation product resulted in brighter strawberries after 5 days storage. The decrease in saturation represented a lower color purity, which was also a negative contribution for strawberry preservation. It was generally caused by the faster rate of anthocyanin degradation compared with its biosynthesis rate in strawberry. Exogenous GABA treatment has been reported to induce anthocyanins accumulation in plants (Zhu et al., 2019). This might explain why the F44/GadB1C1 group showed the slightest change of *C*-value. The changes in *H*-value after 5 days storage for all the samples were overall quite subtle compared to *L* and *C*-values. The differences in Δ*H* among the samples were not significant. This indicated that all the treatments showed no efficacy in obtaining more reddish strawberry. These results demonstrated that treatment with F44/GadB1C1 fermentation products could achieve similar or even better storage performance than sodium benzoate and potassium sorbate for postharvest storage of strawberry.

## Conclusion

The present work reported for the first time the construction of an engineered *L. lactis* strain capable of co-producing nisin and GABA. The two-stage pH-control fermentation strategy enabled further increase in GABA yield as well as alleviation of nisin degradation. Treatment with the freeze-dried product of F44/GadB1C1 fermentation broth was proved to be an effective method to maintain the quality and prolong the storage time of pork and strawberry. Considering the GRAS status of nisin and GABA, the F44/GadB1C1 fermentation products have potential as a natural and safe alternative to synthetic preservatives for meat and fruit. It should be noted that the present study was a first preliminary attempt to construct a nisin and GABA co-producing strain and demonstrated the potential of its fermentation product as a natural and safe preservative for meat and fruit. Further studies, such as integration of GABA biosynthesis genes into the genome and adaptive laboratory evolution, should be conducted to avoid the possible risk of introduction of antibiotic resistance genes in food.

## Data Availability Statement

All datasets generated for this study are included in the article/Supplementary Material.

## Author Contributions

JQ and JL designed the experiments. FM, EN, and GZ carried out the experiments. JL, YD, HZ, and QC analyzed the experimental results. JL, FM, and YD wrote the manuscript. ZZ and JQ revised the manuscript.

## Conflict of Interest

The authors declare that the research was conducted in the absence of any commercial or financial relationships that could be construed as a potential conflict of interest.

## Footnotes

1 http://www.kazusa.or.jp/codon/

2 http://www.yuexukeji.cn/Products-24726616.html
