# Supplementary material for: Co-production of Nisin and γ-Aminobutyric Acid by Engineered Lactococcus lactis for Potential Application in Food Preservation
Source: Front Microbiol. 2020 Jan 29;11:49. doi: 10.3389/fmicb.2020.00049 (PMC7000361; doi:10.3389/fmicb.2020.00049)
Supplement: Supplementary file 1 [file Data_Sheet_1.pdf]

Supporting Information

Supplemental Figure S1

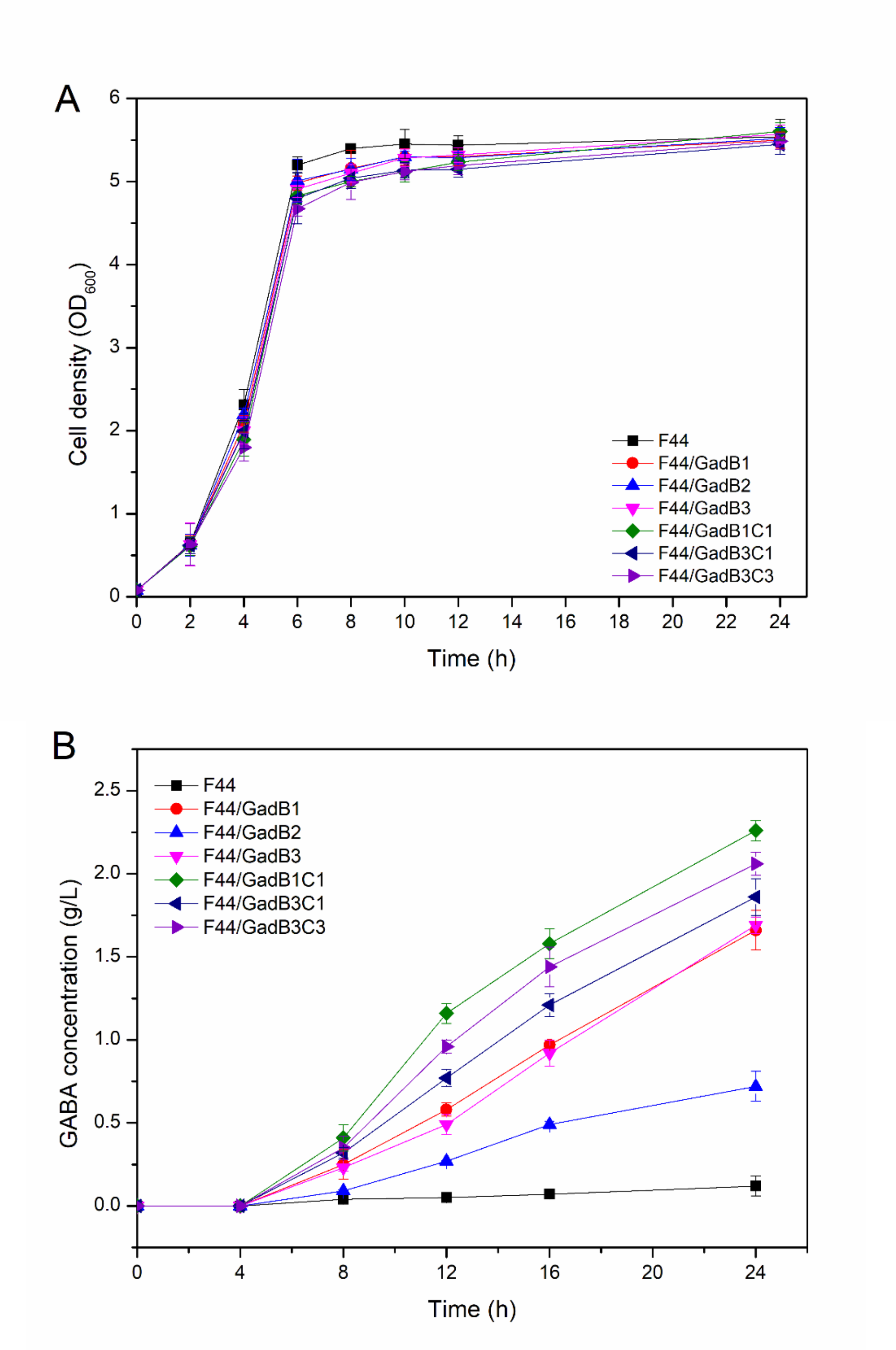

Supplemental Figure S1 Time profile of nisin and GABA production of F44/GadB1C1 in flask fermentation.

Supplemental Figure S2

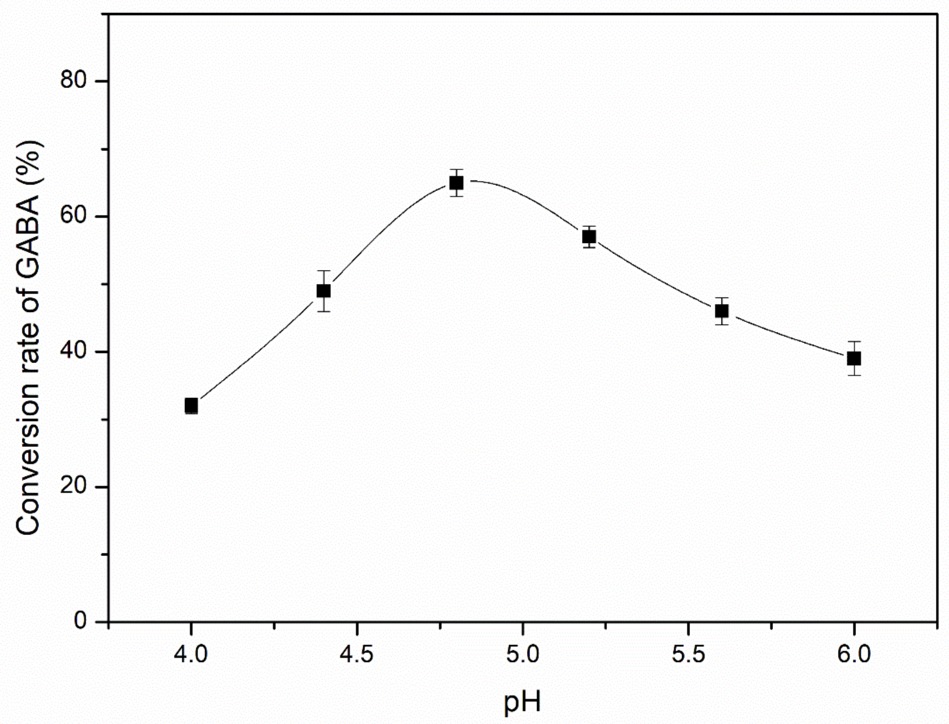

Supplemental Figure S2 Effect of broth pH on conversion rate of GABA of F44/GadB1C1.

Supplemental Figure S3

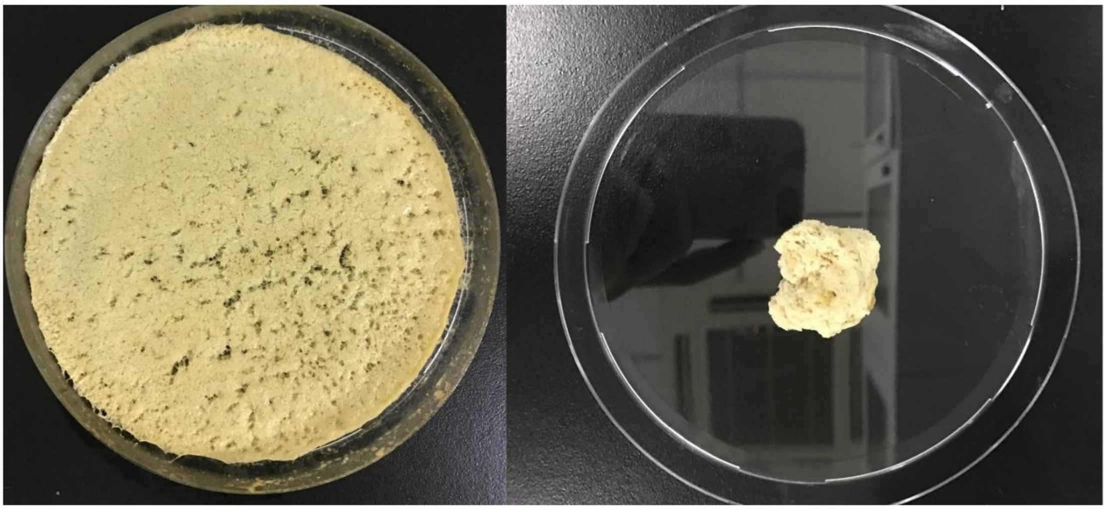

Supplemental Figure S3 Freeze-dried product of F44/GadB1C1 fermentation broth. The product was soft, very friable and slightly sticky. The left picture showed the original shape of the F44/GadB1C1 fermentation broth after dry-freezing and the right picture showed the pinched freeze-dried product in a ball shape.

Supplemental Figure S4

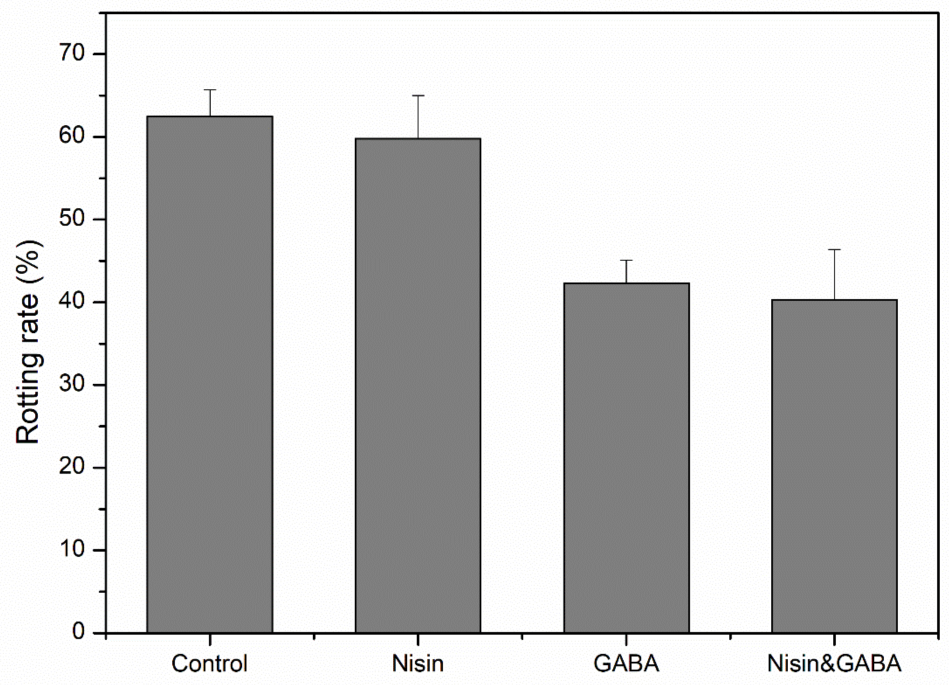

Supplemental Figure S4 Rotting rate of strawberry treated with solution of nisin, GABA and nisin-GABA mixture after 5 days storage at room temperature.

Supplemental Table S1. Strains and Plasmids Used in This Study.

Strains/plasmids	Relevant characteristics	Sources or references
**Strains**		
L. lactis F44	nisin Z producer, constructed through genome shuffling of *L. lactis* YF11	Zhang et al., 2014
L. lactis NZ9000	a non-nisin and non-GABA producing *L. lactis* strain	Zhu et al., 2017
E. coli TG1	used for plasmid preparation	TransGen Biotech (Beijing, China)
M. flavus	used as an indicator strain for bioassay of nisin	ATCC 10240
E. coli MG1655	used for cloning *gadB*	Liu et al., 2019
E. coli TG1/GadB1	*E. coli* TG1 harboring pGADB1	This study
E. coli TG1/GadB2	*E. coli* TG1 harboring pGADB2	This study
E. coli TG1/GadB3	*E. coli* TG1 harboring pGADB3	This study
E. coli TG1/GadB1C1	*E. coli* TG1 harboring pGADB1C1	This study
E. coli TG1/GadB3C1	*E. coli* TG1 harboring pGADB3C1	This study
E. coli TG1/GadB3C3	*E. coli* TG1 harboring pGADB3C3	This study
L. lactis F44/GadB1	*L. lactis* F44 harboring pGADB1	This study
L. lactis F44/GadB2	*L. lactis* F44 harboring pGADB2	This study
L. lactis F44/GadB3	*L. lactis* F44 harboring pGADB3	This study
L. lactis F44/GadB1C1	*L. lactis* F44 harboring pGADB1C1	This study
L. lactis F44/GadB3C1	*L. lactis* F44 harboring pGADB3C1	This study
L. lactis F44/GadB3C3	*L. lactis* F44 harboring pGADB3C3	This study
**Plasmids**		
pLEB124	$Gram^+$ cloning vector with an P45 promoter, Em^r	Qiao et al., 1995
pGADB1	pLEB124 with *gadB* gene from F44, Em^r	This study
pGADB2	pLEB124 with *gadB* gene from MG1655, Em^r	This study
pGADB3	pLEB124 with codon-optimized *gadB* gene from *Lb. buchneri* WPZ001, Em^r	This study
pGADB1C1	pGADB1 with *gadC* gene from F44, Em^r	This study
pGADB2C1	pGADB2 with *gadC* gene from F44, Em^r	This study
pGADB3C1	pGADB3 with *gadC* gene from F44, Em^r	This study
pGADB3C3	pGADB3 with *gadC* gene from *Lb. buchneri* WPZ001, Em^r	This study

Supplemental Table S2. Primers Used for PCR Amplification.

Primer	Primer sequence (5' to 3')[a]
GadB1-F	ATTAACTTAAGTAAGCTTATGTTATACGGAAAAGAAAATCG
GadB1-R	ACGCGTCTGCAGAAGCTTTTAGTGAGTAAAACCATATGTTTT
GadB2-F	ATTAACTTAAGTAAGCTTATGGATAAGAAGCAAGTAACGG
GadB2-R	ACGCGTCTGCAGAAGCTTTCCGTTACCGTTAAACGTTATCAG
GadB3-F	ATTAACTTAAGTAAGCTTATGAGTGAAAAAAATGATGAACA
GadB3-R	ACGCGTCTGCAGAAGCTTAGTTGGCAATGATTTACCA
GadC1-F	ACGTCATATGGATCCATGATGAATCAAAAAAAATTATCA
GadC1-R	CCTATATATGGATCCTTAATGTTTTAAGATATGTTCTTCT
GadC3-F	ACGTCATATGGATCCATGGAAAGAGACGACGTTGA
GadC3-R	CCTATATATGGATCCCTACTTTTTAGCAGTTTCTCCAT

[a] Underlined sequences indicate homologous sequences.

References

1. Zhang, Y. F., Liu, S. Y., Du, Y. H., Feng, W. J., Liu, J. H., and Qiao, J. J. (2014). Genome shuffling of *Lactococcus lactis* subspecies *lactis* YF11 for improving nisin Z production and comparative analysis. *J. Dairy Sci.* 97, 2528-2541. doi: 10.3168/jds.2013-7238.

2. Zhu, D., Fu, Y., Liu, F., Xu, H., Saris, P. E. J., and Qiao, M. (2017). Enhanced heterologous protein productivity by genome reduction in *Lactococcus lactis* NZ9000. Microb Cell Fact, 16, 1. doi:10.1186/s12934-016-0616-2

3. Liu, J., Li, H., Xiong, H., Xie, X., Chen, N., Zhao, G., et al. (2019). Two-stage carbon distribution and cofactor generation for improving L-threonine production of *Escherichia coli*. Biotechnol. Bioeng, 116, 110-120. doi:10.1002/bit.26844

4. Qiao, M, Immonen, T., Koponen, O., and Saris, P. E. J. (1995). The cellular location and effect on nisin immunity of the NisI protein from *Lactococcus lactis* N8

expressed in *Escherichia coli* and *L. lactis*. *FEMS Microbiol. Lett.* 131, 75-80. doi: 10.1111/j.1574-6968.1995.tb07757.x
